# High Potencies and Dr. P. Jousset in Paris

**Published:** 1885-09

**Authors:** B. Fincke

**Affiliations:** Brooklyn, N. Y.


### HIGH POTENCIES AND DR. P. JOUSSET IN PARIS.

B. Fincke, M. D., Brooklyn, N. Y.

*From the Zeitschrift des Berliner Vereins homœopathischer Aerzte, Bd. IV, Heft* 6.

Under the title, "*On the Unlimited Attenuation of Homœopathic Remedies*," an extract of a letter of Dr. P. Jousset, in Paris, to Dr. Gallavardin (*Art Med.*, September, 1884) has been published in the *Zeitschrift des Berliner Vereins homœopathischer Aerzte*, Vol. IV, p. 227, with the supposition that the information about certain methods of potentiation given therein might be of general interest. This supposition, however, finds no justification in the communication, for never has greater ignorance of this important subject been displayed than in this letter of Dr. Jousset. With perfect justice, its tendency has not been touched upon by the editor, nevertheless I would like to recur to it briefly, since here is said quite unconcernedly: "*This sect, the adherents of infinitesimal doses into the infinite* (*sic*), *is a true ballast for us which must be thrown overboard. It deceives itself and the medical world when it talks about high attenuations.*"

Let us now consider who of the homœopathic fraternity are to be understood to belong to that sect according to the Frenchman's withering critique.

First and foremost, *Hahnemann* himself, the intrepid pioneer of that sect (he once spoke of the pernicious mixing-sect, but in our days his dictum is reversed), who decidedly made the cure dependent upon the properly potentiated dose, which must have nothing physical or chemical about it.

Then *Gross*, *Stapf*, *Bœnninghausen*, *Hering*, *Aegidi*, *Joslin*, *Dunham*, *Nuner*, and others. The life of all these champions for the pure Hahnemannian lore lies closed before us. They shine as stars of the first magnitude in the homœopathic firmament. They have left their experience for the now living as a legacy, not an inconsiderable number of whom belong to the most able, learned, and active adherents of Homœopathy, and their names need not be mentioned, being known all over the world. To be in such society is an honor which Dr. Jousset cannot share, not even estimate, though unconsciously he, in his condemnation of honorable men, needs must tell the truth. For when comparing Homœopathy with a ship it certainly carries, in the weight of the pure doctrine and of an honorable execution of the same in the deeds of their adherents, the true ballast in its hold which secures to the ship its steady course and a safe equilibrium. If *that* ballast is thrown overboard the ship must surely topple over and go down.

Let us now examine where the deceit is of which Dr. Jousset is talking. One would think he must be well informed, because he talks with such assurance, but how much are we disappointed! He even does not speak from personal experiment, but refers to the apothecary.

First comes the preparation of *Jenichen's* high potencies (not Jennichen). It is said that Jenichen had prepared the fourth potency and moistened globules with it, which he shook violently. To these globules he added one drop of alcohol, and shook it again. Every shaking stroke was called an attenuation. Consequently his forty thousandth is only the fourth to which a few drops of alcohol were added, and which for some time was powerfully shaken.

Every homœopathician who has prepared his own remedies will see at a glance that this is pure nonsense, and, like all nonsense, perfectly without any foundation in fact.

The mode of Jenichen's preparation is contained in a letter of Jenichen to Dr. Hering, an extract of which has been published in the *Organon* of Dr. Skinner, Vol. II, p. 429 (Liverpool, Holden, 1879). From this it appears that Jenichen wanted to carry up the twenty-ninth centesimal potency of Plumb. ac., but found the tincture dried up and the cork shriveled and loose in the neck of the vial. Curious to know whether there was any virtue left in the empty vial, he filled it three-quarters full of alcohol and shook it. Of this tincture he took one drop, added it to three hundred drops of alcohol (70–80° Richter), and prepared from it the three centesimal two hundredth potency in a potentiating vial of four-and-a-half inches in length and one-half ounce in weight. A case of perspiration of the soles of the feet, smelling like foul cheese, ever since the second year of life in a young, athletic fellow, six feet high and of twenty years of age, offered an opportunity of testing the new remedy. Jenichen put a few pellets of said Plumb. ac.$^{200}$ (three centes.) in a vial and allowed the patient to take one full inhalation from it. The result was a perfect cure within ten days. Jenichen considered this as a revelation, and from that time made all his high potencies from evaporated tinctures in the following manner: The potencies were grafted from the twenty-ninth centesimal potencies in the way described above, and carried up to eight hundred in the ratio of 1 : 300 with twelve shaking strokes. But from nine hundred he potentiated in the ratio of 2 : 12,000 with thirty shaking strokes, but for every four hundred degrees more he added twelve strokes to each potency. These highest potencies had been made with the clearest water of the Schwerin Lake. The potentiating vial weighed eighteen ounces, including the contents. The fluid at every stroke gave a shrill sound like the tinkling of silver money.

Hence there is not the least doubt that Jenichen did *not* count the shaking strokes for potencies, but that the potencies were real dilutions or attenuations according to Jenichen's peculiar scale, viz.:

1 : 100 up to the twenty-ninth,
1 : 300 from twenty-nine to eight hundred,
2 : 12,000 from nine hundred to forty thousand, the highest potency which came from his laborious hand.

It has become the fashion to put down Jenichen, and to slander him in every way, though he was a man of honor and moral worth, a man scientifically educated, a veterinary physician approved of by his government, and appointed by the same as examiner in that quality, and besides an excellent homœopath who knew the materia medica pura as well as anybody at his time. Therefore, honor be paid to the man to whom honor is due. For he has given his very life for the great cause of potentiation and for Homœopathy. If the horses could speak, they would proclaim his praise, and would in this age of monuments raise one for him to the sky; for since Jenichen they are being cured with high potencies as well as men.

Everybody can now easily judge of the value of Dr. Jousset's baseless assertion. It is true, that the high potencies of Jenichen have been used by the most prominent homœopathicians in practice with the greatest benefit up to the present day.

Now comes the turn of *Dr. Fincke* (not Finke), whose high potencies are declared to be merely *nothing*, though already excellent provings and cures with not only the thirtieth to CM, but also with the five and six millionth centesimal potencies of Lachesis are extant. But the learned Dr. Jousset has unwittingly and unluckily criticised not Fincke's potentiating vial, but that in Bœricke's machine, which has no bottom to it, and Mr. Bœricke has to thank him for calling his millions which have been said to have been effective in the hands of several physicians, *nothing*. Dr. Fincke's vial has, like every well-conditioned vial and every good head, its opening at the neck. Dr. Fincke also is not responsible for the elegant expression of "Bottle-washing," which has been invented by the embittered enemies of high potencies for other fluxion preparations, and in this relation indeed not without some justification. Dr. Fincke calls his method the fluxion-method. On this method one could not wash a dirty bottle clean even if a thousand tons of clean water were run through it. Fincke prepared his initial potencies centesimally up to the sixth (resp. thirtieth) by hand, but even these are called *nothing* by Dr. Jousset. These initial potencies are submitted to gentle fluxion in the ratio of 1 : 100 to infinity—for seven millions is so far by experiment finity—so that by the quiet undulations the thorough mixture of the medicine with the flowing vehicle without intervention of the manipulator is insured. By this process not the medicament alone, as Dr. Jousset teaches, is removed from the vial, but also the mass of vehicle interpenetrated by it, or else no fluxion and consequently no potentiation could take place, because, as one philosopher has wisely remarked, a full vessel cannot be made fuller. Only those potencies are taken from the fluxion which are to be preserved, for who could find room and means for seven millions of potencies? The fluxion proceeds forward and the potentiation backward, and the potencies obtained are pushed out above. The Hahnemannian element of shaking is replaced by the gentle undulation of the flux, and the potencies thus obtained can aptly be called *fluxions* because they correspond with the Newtonian conception of a fluxion, which, though being infinitesimal, yet has a definite value. Hence a six millionth is still a finite potency because its action on the organism can be clearly observed, and inasmuch as an extreme minuteness is finite, be it ever so inaccessible to quantity, it loses the quality of infinitesimality and remains only a *minututum*.

The fact alone that high potencies thus prepared are efficacious in proving and healing according to homœopathic principles must enervate the objections of the physicists. In Dr. Buchmann's proving of the six millionth fluxion-potency of Lachesis, forty-two symptoms of Dr. Hering's provings with the thirtieth have been verified. One would think that throughout the whole series of six million potencies the medicament must have been intimately mixed with the vehicle, as Jousset expresses it, or it would have been "washed" out long before, and nothing could have been left at such an altitude which could have produced such an extraordinary proving as the world has never seen before.

Also the much bedraggled *Korsakoff* is accused of bottle-washing, though this could hardly have been his intention when—alas! fifty-three years ago—he communicated his famous discoveries for the first time. This noble man, though a noble man whose disinterested and laudable endeavors in the field of homœopathics have been properly estimated in the history of Homœopathy in Russia by Dr. C. Bojanus, soon found that the Hahnemannian method was not practicable for higher attenuations, and conceived the judicious idea of reversing the process. If, as Dr. Jousset teaches, the potentiating vial is merely emptied by putting it upside down, indeed, a greater quantity of vehicle will adhere to the walls than is desirable for the correctness of the scale. If, however, the vial is well emptied by jerking the fluid out, a residue can be obtained which agrees very well with the Hahnemannian scale. Hahnemann has sanctioned this process when, in a letter to Stapf (*Archiv* xi, 3, p. 106), he writes: "For preparations of so enormously highly potentiated attenuations of medicinal substances the process of the noble Korsakoff is as ingenious as to the point. On testing it with very sensitive balances it will be found that a vial of the given size, after powerful squirting out of one hundred grains of water contained in it, retains almost exactly one grain of water adhering to its walls, a circumstance which makes the further attenuations very safe and trustworthy, so that nothing can be brought against it, and which simplifies and eases the operation incredibly."

Hence the Korsakoffian method cannot be reproached with more incorrectness than the Hahnemannian itself. For the latter is also only conventionally exact. According to it one grain is deemed equal to one drop. The fourth attenuation is made by placing one grain of the third trituration into a mixture of fifty drops of water and fifty drops of alcohol, which gives drops of a different size and a different ratio. And even the size of the drops varies with the vials according to the form of the lip and of the strength of the alcohol. Besides, the dropping over of single drops from vials made from glass-tubes is an impracticable thing. For the purpose of higher potentiation, therefore, the Korsakoffian method is as exact as it can and need be without encroaching upon the practical and scientific necessity. Every expert in this matter must admit that what is understood as Hahnemannian exactness, which, as above shown, does not exist, is unattainable in a process dealing with such enormous masses of vehicle, and with medicine of an incredible subtlety. It would involve a reprehensible purism which, in the conceit that the potencies as far as the millions could be prepared with mathematical precision, deprives itself of the benefit of these remedies by means of which many cases can be cured which remain untouched or are only changed to other diseases by the crude substances and low potencies.

Of the contact potencies of Korsakoff, this most remarkable discovery, which has even a greater bearing in its consequences than that of his dilution-potencies, Dr. Jousset has nothing to say. But we learn that he has not even made experiments himself, but Mr. Catellan claims his sympathy on account of the labor and patience which it cost him to prepare a potency as high as—just think of it—the two hundredth. Of the many other industrious men who have made high potencies on the Korsakoffian plan, *e. g.*, Lehrmann, with his two hundredth, which were preferred by Dr. Bœnninghausen, and have gone over the world, of Dunham's two hundredth, which are highly estimated here, Dr. Jousset knows nothing, nothing of Tafel's five hundredth or one thousandth, which likewise were made after Korsakoff's plan with alcohol, not to speak of many others.

The argumentation of Jousset, which rests upon the statement of an experienced pharmacist, and intends to show the absurdity and impracticability of high potentiation on the Hahnemannian plan, serves only to prove quite the contrary of his intention, viz.: to show that high potencies cannot be prepared. For, if the Hahnemannian method is impracticable, another method is indicated, and if this one solves the problem of high potentiation without the great outlay in vials, alcohol, labor, and other expenses, why—"*mein Liebchen was willst du noch mehr?*"

Those who cannot get over the Hahnemannian method, upon which the methods of Korsakoff and Fincke are based, since they adopt exactly the ratio of 1 : 100, are indeed like the hypnotized hen, which cannot get away from the line made with chalk from along its bill upon the table.

This may suffice to repel the groundless assertion of Dr. Jousset that those who use the ten-thousandth or a higher potency have only the fourth Hahnemannian or nothing, and that their successes are by no means out of a level with those which in general are gained by Homœopathy. This stratagem to stamp the high potencies as low ones, and thereby to explain their efficacy in curing disease, has been tried before on this continent.

But this effort to help macrodosia on its legs has been futile, in the face of the many most interesting cases of the greatest variety cured in this country and in Europe during the last forty years and more.

In looking at the efforts of our opponents to put down high potencies at any price, nothing is more depressing than the discovery that they have not even advanced as far as Korsakoff (1831). What prevents them, if they have real scientific progress at heart, to select a remedy from our materia medica and to subject it to the process of potentiation according to Korsakoff, and to test the product thus gained by their *own* exertion upon healthy and sick people according to homœopathic law? That is the only way to arrive at a result consonant with the exactions of science. Then will the fluxion potencies also find favor in their eyes. When shall they cease to attack those who, passing beyond what is known and following it up, try to find new ways and means in order to make the healing of man and beast still safer and easier than it is now? Again, when shall the opponents make up their minds by their own experiment to convince themselves that the high potencies are no more low attenuations, nor any attenuations at all, but just potencies, forces, medicinal forces, which, by the potentiating process, are unfolded and developed, as maintained by Hahnemann repeatedly and with perfect justice? They surely cannot kill anybody with a hundred-thousandth potency, but cure many a knotty case, as appears from the wonderful Phosphorus cure of Dr. Buchmann—see HOMŒOPATHIC PHYSICIAN, August, 1884—a true model-cure after Hering. There is no danger, not even the danger of ridicule, which indeed many fear more than death. One must get over it. Truth must always remain truth.

*Ceterum censeo, macrodosiam esse delendam.*

